# Mitochondria‐targeted antioxidant MitoQ reduced renal damage caused by ischemia‐reperfusion injury in rodent kidneys: Longitudinal observations of T_2_‐weighted imaging and dynamic contrast‐enhanced MRI

**DOI:** 10.1002/mrm.26772

**Published:** 2017-06-12

**Authors:** Xiaoge Liu, Michael P. Murphy, Wei Xing, Huanhuan Wu, Rui Zhang, Haoran Sun

**Affiliations:** ^1^ Department of Radiology Tianjin Medical University General Hospital Tianjin China; ^2^ Medical Research Council Mitochondrial Biology Unit, Cambridge BioMedical Campus Cambridge UK; ^3^ Department of Radiology Third Affiliated Hospital of Suzhou University Changzhou Jiangsu China.

**Keywords:** kidney, renal function, ischemia‐reperfusion injury, IRI, dynamic contrast‐enhanced magnetic resonance imaging (DCE‐MRI), histopathology

## Abstract

**Purpose:**

To investigate the effect of mitochondria‐targeted antioxidant MitoQ in reducing the severity of renal ischemia‐reperfusion injury (IRI) in rats using T_2_‐weighted imaging and dynamic contrast‐enhanced MRI (DCE‐MRI).

**Methods:**

Ischemia‐reperfusion injury was induced by temporarily clamping the left renal artery. Rats were pretreated with MitoQ or saline. The MRI examination was performed before and after IRI (days 2, 5, 7, and 14). The T_2_‐weighted standardized signal intensity of the outer stripe of the outer medulla (OSOM) was measured. The unilateral renal clearance rate k_cl_ was derived from DCE‐MRI. Histopathology was evaluated after the final MRI examination.

**Results:**

The standardized signal intensity of the OSOM on IRI kidneys with MitoQ were lower than those with saline on days 5 and 7 (*P* = 0.004, *P* < 0.001, respectively). K_cl_ values of IRI kidneys with MitoQ were higher than those with saline at all time points (*P* = 0.002, *P* < 0.001, *P* = 0.001, *P* < 0.001). Histopathology showed that renal damage was the most predominant on the OSOM of IRI kidneys with saline, which was less obvious with MitoQ (*P* < 0.001).

**Conclusions:**

These findings demonstrate that MitoQ can reduce the severity of renal damage in rodent IRI models using T_2_‐weighted imaging and DCE‐MRI. Magn Reson Med 79:1559–1667, 2018. © 2017 The Authors Magnetic Resonance in Medicine published by Wiley Periodicals, Inc. on behalf of International Society for Magnetic Resonance in Medicine. This is an open access article under the terms of the Creative Commons Attribution License, which permits use, distribution and reproduction in any medium, provided the original work is properly cited.

## INTRODUCTION

Nephron‐sparing nephrectomy has been accepted as the standard surgical procedure for the treatment of small renal tumors and in patients with bilateral tumors 1, 2. Temporary renal artery occlusion is essential during the operation to prevent intraoperative bleeding and to provide a bloodless surgical field 3. Hence, warm ischemia and subsequently renal ischemia‐reperfusion injury (IRI) are inevitable and are a major cause of postoperative renal functional decline 4, 5. Kalogeris et al. 6 proposed that pharmacological preconditioning could contribute to the activation of cell survival mechanisms and protection of the kidneys from IRI. The search for novel medical interventions is of great research interest 7. Cell death programs activated by ischemia and reperfusion are related to apoptosis, necrosis, autoimmune responses, and enhanced reactive oxygen species (ROS) generation, and there might be a reciprocity between the programs and mitochondrial oxidative damage in the early phases of IRI 8, 9. The oxidative stress caused by excessive amounts of ROS during IRI could alter mitochondrial oxidative phosphorylation and activate membrane phospholipids proteases, and more importantly, oxygen free radicals during reperfusion could result in lipid peroxidation 10. As mitochondrial oxidative damage is central to IRI damage, decreasing mitochondrial oxidative activity during IRI could be a potential therapeutic approach. Because untargeted cellular antioxidants cannot accumulate in mitochondria and they are not protective 11, Murphy et al. developed the mitochondria‐targeted antioxidant MitoQ. MitoQ is conjugated to a lipophilic cation that can rapidly pass through the mitochondria phospholipid bilayer and accumulate within the mitochondria with the help of the electrochemical gradient 11.

Magnetic resonance imaging can provide excellent morphologic and functional information for evaluating renal pathophysiology noninvasively in vivo, and is suitable for repeated measurements. The differences between T_2_ relaxation times for different tissues enable MRI to be used in the evaluation of morphologic changes with high spatial resolution 12. The severity of renal IRI damage varies in different regions. Hueper et al. demonstrated that the T_2_ values of the outer stripe of the outer medulla (OSOM) could be used to distinguish between severe injury and moderate injury after IRI in mice 13. This capability probably exists because the thick ascending limb of Henle's loop and the S3 segment of the proximal tubule in the OSOM consume far more oxygen than any other parts to achieve sodium balance involving active reabsorption 14. It has been well documented that renal functional parameters obtained from dynamic contrast‐enhanced MRI (DCE‐MRI) are closely correlated with radionucllde renography parameters 15. Baumann and Rudin proposed that the first‐order rate constant k_cl_, which reflected tracer transportation from the renal cortex to the medulla in the initial phase of DCE‐MRI, might provide a good estimation of the kidney clearance rate 16. Laurent et al. 17 further demonstrated that k_cl_ data estimated by DCE‐MRI correlated well with glomerular filtration rate values assessed using the inulin clearance assay. In this setting, the left renal artery was clamped and the right kidneys were kept as controls, which made this variable ideal for comparisons to reveal renal morphologic and functional changes bilaterally in living animals. It would be beneficial, both methodologically and ecologically, if the quantitative estimations of T_2_ signal intensities of the OSOM and k_cl_ values obtained from DCE‐MRI before and after IRI could noninvasively reveal the severity of renal damage, and be used to evaluate the effect of medical pretreatment in protecting against IRI.

The aim of the study was to investigate the effect of MitoQ in reducing the severity of renal damage caused by IRI in rats based on the longitudinal observations of T_2_‐weighted imaging and DCE‐MRI with quantitative estimations of unilateral renal dynamic parameters.

## METHODS

### Animal Preparation

This study was approved by the local Animal Care and Use Committee. Twenty male Sprague–Dawley rats (Beijing University Health Science Center) with an average weight of 250 g were used in this experiment. The rats were randomly divided into four groups: Group I (IRI with MitoQ treatment, n = 5), in which the rats were administered with MitoQ (donated by Antipodean Pharmaceuticals Inc: 2.8 mg/kg in 700 µL 0.9% saline) 15 min before the onset of warm ischemia via the tail vein at an injection rate of 20 µL/s, then subjected to left renal ischemia for 45 min followed by reperfusion; Group II (IRI with saline treatment, n = 5), which underwent the same procedure as Group I except that the injectant was 700 µL 0.9% saline instead of MitoQ; Group III (normal kidneys with MitoQ treatment, n = 5), in which the rats were administered with MitoQ in the same way as in Group I but without the IRI procedure; and Group IV (normal kidneys with saline treatment, n = 5), in which the rats were administered with saline in the same way as in Group II but without the IRI procedure.

### Ischemia‐Reperfusion Injury Model

The animals were fasted overnight with free access to water before the operation. They were placed on a homoeothermic pad to maintain body temperature at 37 ± 1°C. Anesthesia was maintained by mask inhalation of isoflurane vaporized at concentrations of 1 to 2.5% during the operation. The abdominal region was shaved and sterilized. After a midline laparotomy was performed, the left renal artery was isolated and clamped using microvascular clips to reversibly occlude renal blood supply. Complete occlusion was verified by observing the color of the kidney, running from bright red to reddish brown color. A warm ischemia time (WIT) of 45 min was chosen on the basis of previous experience to ensure renal function decline and to minimize the mortality of the animals. After the clips were removed when the WIT was up, the kidneys were observed as returning to bright red color, and then the incision was sutured. After the operation, the rats were returned to their cages in a temperature and humidity‐controlled facility with a constant 12‐h light/dark cycle.

### MRI Examination

The MRI scanning was performed before the operation (day 0) and on days 2, 5, 7, and 14 after IRI on a 3T scanner (MR750, GE Healthcare, Chicago, IL) with a 6‐cm internal diameter four‐channel phase‐arrayed animal coil (Magtron, Shenzhen, China). Rats were fasted but free of drinking 2 h before the MRI examination, and anesthesia was maintained by mask inhalation of isoflurane vaporized at concentrations of 1 to 2.5% during the scanning. Each animal was placed in a supine position, and appropriate compression was applied to the abdominal region to restrict the respiration movement to a minimal extent.

Coronal high‐resolution T_2_‐weighted imaging (fat‐suppressed fast recovery fast spin echo, repetition time/echo time 3106 ms/92 ms; matrix size 352 (frequency) × 352 (phase); echo train length 22; field of view (FOV) 6.0 × 6.0 cm; slice thickness 1.5 mm; slice spacing 0.5 mm; bandwidth ± 46.446 kHz, two signal averaged, acquisition time 4 min 13 s) and DCE‐MRI (3D liver acceleration volume acquisition, repetition time/echo time 5.3 ms/2.0 ms; flip angle 15°; bandwidth ± 125 kHz; matrix size 160 (frequency) × 128 (phase); frequency FOV 13.0 cm; phase FOV 0.80; slice thickness 2 mm; spacing 0 mm) were acquired. For DCE‐MRI, a precontrast data set was obtained, after which a bolus injection of 0.04 mmol/kg of diluted gadolinium‐diethylenetriamine pentaacetic acid (Gd‐DTPA) solution (100–120 µL; Magnivist, Bayer‐Schering, Berlin, Germany, 125 mmol/L) was administered through a catheter in the tail vein at an injection rate of 100 µL/s. The DCE‐MRI scanning was repeated immediately after the administration of Gd‐DTPA. A total of 12 coronal slices were acquired in 3 s, and 20 enhanced phases were continuously obtained in 60 s.

### Relationship between the Signal Intensity and Gd‐DTPA Concentration

It has been documented that the signal intensity (SI) changes can be converted into longitudinal relaxation rate (R1) changes using a polynomial equation 18, and R1 correlated well with the concentration of Gd‐DTPA 19. Moreover, there is a linear relationship between SI changes and the increased concentration of Gd‐DTPA between 0 and 4 mmol/L 20. In the present study, the estimated Gd‐DTPA concentration in the renal parenchyma was less than 4 mmol/L because of the low administration dose; therefore, it was feasible to reflect the Gd‐DTPA concentration based on the SI.

### Image Analysis and k_cl_ Calculation

**Figure 1:**
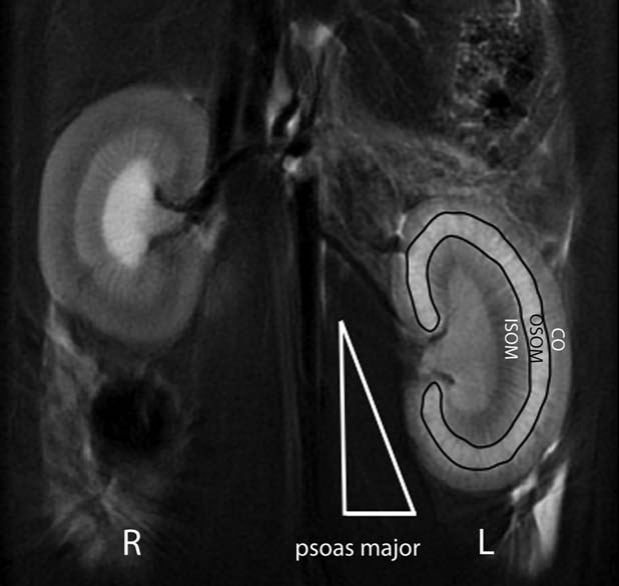
Example of ROI plotting on coronal fat‐suppressed T_2_‐weighted imaging after IRI. The CO, OSOM, and ISOM are illustrated on the left renal parenchyma. The ROI of the entire OSOM on a central section of the left kidney is delineated by a black line; the left psoas major is delineated by a white triangle.

The SI changes were evaluated on an ADW4.5 GE workstation. The SI of the renal cortex (CO), the outer stripe of the OSOM, the inner stripe of the outer medulla (ISOM), and psoas major on the middle section of bilateral kidneys were inspected on coronal T_2_‐weighted imaging. The regions of interest (ROIs) of the OSOM and homolateral psoas major were delineated (Fig. 1). The renal SI was prone to be affected by the adjacent tissues (intra‐abdominal fat or gas in the gastrointestinal tract), whereas the SI of the psoas major was relatively constant. Therefore, a T_2_‐weighted standardized signal intensity (SSI) was obtained by calculating the ratio of the SI of the OSOM and the homolateral psoas major.

**Figure 2:**
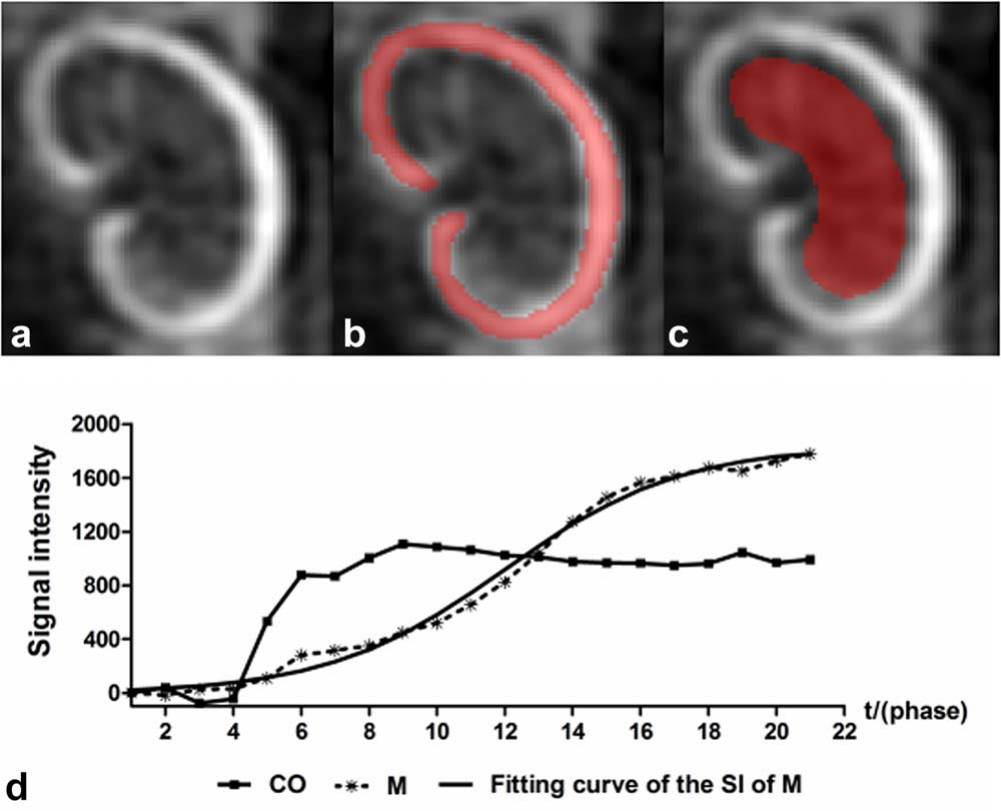
Example of renal semi‐automatic segmentation and ROI setting on DCE‐MRI, the TICs of the renal CO and the M, and the fitting curve of the SI of the M. **a**: Original contrast‐enhanced image of the left kidney. **b**: The ROI of the CO was recognized automatically and is rendered in red. **c**: The ROI of the M was delineated manually and is shown in red; please note that the ROI of the M was carefully separated from the CO to avoid the partial volume effect. **d**: The TICs of the renal CO, M, and the fitting curve of the SI of the M. The TIC of the CO rose radically in the early phases until reaching the platform, whereas the TIC of the M kept rising gradually for a long time. The fitting curve of the SI of the M was a smooth curve overlapping the true TIC of the M. Note that the acquisition time in each phase was 3 s.

The DCE‐MRI data were analyzed using locally developed software (Northeastern University, Shenyang, China) on a HP workstation (Palo Alto, CA). The DCE images were segmented and measured semi‐automatically. First, the most optimal phase, in which there was a clear corticomedullary demarcation between the CO and the whole medulla (M, including OSOM, ISOM, and the inner medulla (IM)), was manually selected among the multiphasic images (Fig. 2a); then, the ROIs of the entire CO and M regions were drawn and copied to the other phases. The ROI of the M was carefully separated from the CO to avoid the partial volume effect (Figs. 2b and 2c). Second, the average SI of the CO and M were calculated, and the absolute SI in each phase was obtained by multiplying the area of the ROI and the slice thickness. The relative SI was determined by subtracting the SI of the precontrast image from the absolute SI. Third, the time signal–intensity curves (TICs) of the CO and the M were plotted and the fitting curve of the SI of the M was determined (Fig. 2d).

The clearance rate constant k_cl_ was obtained using the mathematical model proposed by Baumann and Rudin 16, and k_cl_ was represented in the following equation:

$$k_{cl}=\left(dc_{m}/dt\right)/c_{c}\left(t\right)$$

where Cc is the cortical Gd‐DTPA concentration; Cm is the whole medullar Gd‐DTPA concentration; dc_m_/dt is the slope of the TIC of the medullar, representing the uptake of Gd‐DTPA in urine; c_c_ is the cortical tracer concentration, closely resembling the vascular Gd‐DTPA concentration; and t is the time, determined when there was the largest discrepancy between CO and M on DCE images; it ranged from 12 to 18 s in the study and varied in each individual.

### Histological Examination

All rats were sacrificed after the final MRI examination, and both kidneys were harvested for histopathological examination. The samples were fixed with 5% buffered formalin, embedded in paraffin, and then cut into 5‐mm‐thick sections. The sections were stained with hematoxylin and eosin, and the microscopic changes of each sample were examined by an experienced pathologist who was blinded to the grouping. The alterations in renal tubules, including tubular dilation, epithelial cell vacuolization, desquamation and cast formation, as well as alterations of the interstitial tissue, including interstitial tissue edema and inflammatory cell infiltration, were demonstrated. Renal damage was scored using a semi‐quantitative method based on the injury area of involvement with a scale of 1 to 4 as follows: 1, absence of injury; 2, localized injury ( < 25% renal injury); 3, extended injury ( < 50% renal injury); and 4, severe injury ( > 50% renal injury) 21.

### Statistical Analysis

The statistical analysis was performed with Statistical Product and Service Solutions (SPSS) software 12.0.1 (IBM Corp, Armonk, NY). Data were presented as mean ± standard deviation for a normal distribution and median ± Q for a nonnormal distribution. The Kolmogorov‐Smirnov test was applied to assess normally distributed data, and Levene's test was used for the variance homogeneity assay. Comparative analyses of SSI and k_cl_ values were performed using the least significant difference (LSD) at different time points. Kruskal‐Wallis test and Mann‐Whitney test were used for pathological damage score analyses. A *P*‐value of less than 0.05 was taken as a significant difference.

## RESULTS

**Figure 3:**
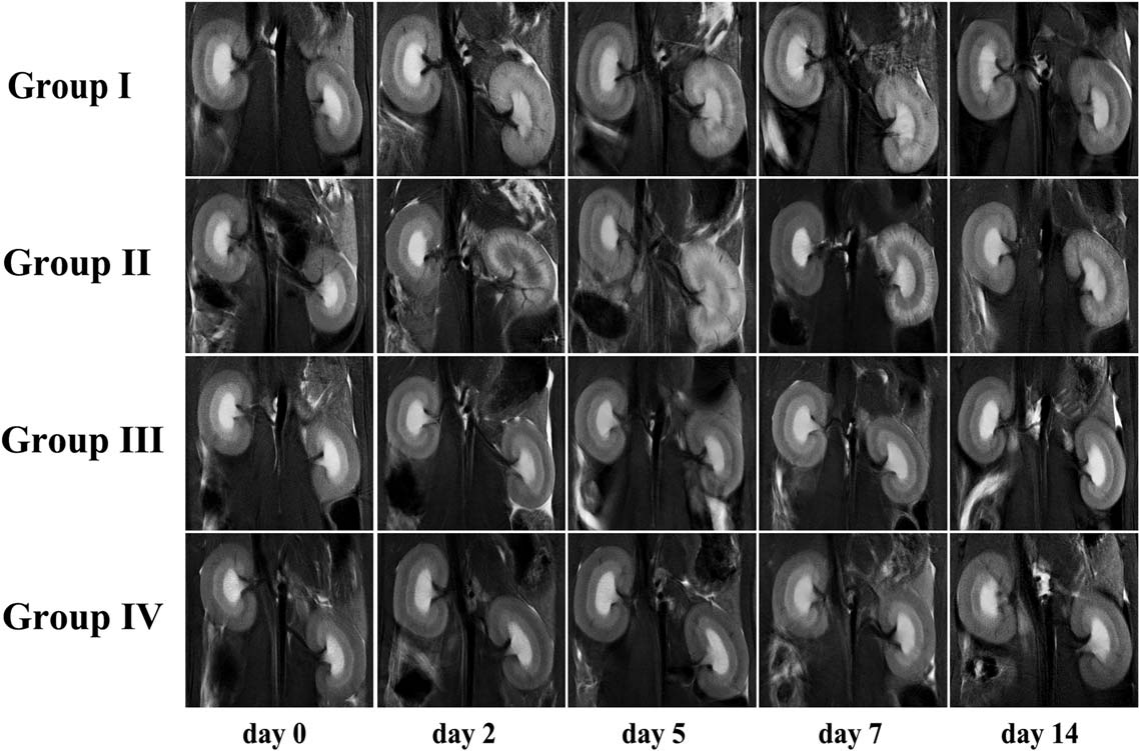
Examples of fat‐suppressed T_2_‐weighted images in the central coronal section of the bilateral kidneys, before (day 0) and after IRI (days 2, 5, 7, and 14), in the four groups. The OSOM of the left kidneys began to appear as a hyperintense stripe on day 2 in Groups I and II, with distinct boundaries that radiated to the renal cortex, and then broadened with obscure boundaries and were even brighter thereafter on day 5. Then, they began to shrink but still had a hyperintense signal on day 7. Subsequently, the OSOM returned to normal size with residual bright strains. The hyperintense stripe of the OSOM in Group II was much brighter than that in Group I. No notable changes were observed on the right kidneys in the four groups and the left kidneys in Groups III and IV at all time points.

Coronal T_2_‐weighted imaging showed serial SI changes on the bilateral kidneys in four groups before (day 0) and after IRI (days 2, 5, 7, and 14 ) (Fig. 3). In healthy kidneys (day 0), CO and OSOM demonstrated similar moderate signal intensity and ISOM showed a relatively hyperintense signal. After the IRI procedure, the left OSOM in Groups I and II began to appear as a hyperintense stripe (day 2) with distinct boundaries that radiated to the CO, and then broadened with obscure boundaries and were even brighter thereafter (day 5). Then, they began to shrink but still showed a hyperintense signal (day 7). Subsequently, the OSOM returned to normal size with residual bright strains. The hyperintense stripe of the OSOM in Group II was much brighter than that in Group I, which indicated more severe edema and worse injury in Group II. No notable changes could be observed in the left kidneys in Groups III and IV and the right kidneys in all four groups at all time points.

**Figure 4:**
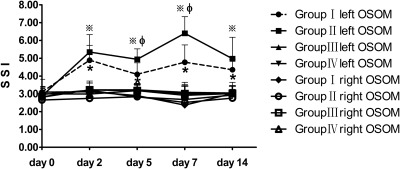
Graphs of SSI of the OSOM on bilateral kidneys in the four groups before (day 0) and after IRI (days 2, 5, 7, and 14). The SSI of the bilateral OSOM on day 0 were not significantly different among the four groups. The SSI of the left OSOM in Groups I and II rose sharply on day 2, and were higher than those of the other kidneys (the right kidneys in all of the groups and the left kidneys in Groups III and IV) at all time points after IRI, which fluctuated within a narrow range. The SSI of the left OSOM in Group I on days 5 and 7 were lower than those in Group II (*P = * 0.004, *P < * 0.001, respectively). No significant differences in SSI existed between any two of the other kidneys. Significant differences of SSI of the OSOM between the left kidneys in Group I and the other kidneys are indicated as * (*P < * 0.05); those between the left kidneys in Group II and the other kidneys are indicated as ※ (*P < * 0.05); and those between the left kidneys in Group I and the left kidneys in Group II are indicated as ※ (*P < * 0.05).

The average SSI of the bilateral OSOM in the four groups are listed in Table 1. The SSI of bilateral OSOM on day 0 were not significantly different among the four groups. The SSI of the left OSOM in Groups I and II rose sharply on day 2, and they were higher than those of the other kidneys (the right kidneys in all four groups and the left kidneys in Groups III and IV) at all time points after IRI, which fluctuated within quite a narrow range. The SSI of the left OSOM in Group I on days 5 and 7 were lower than those in Group II (*P* = 0.004, *P* < 0.001, respectively). No significant differences in SSI existed between any two of the other kidneys (Fig. 4).

**Table 1:** Average SSI (Mean ± Standard Deviation) of the OSOM on Bilateral Kidneys, Before (Day 0) and After IRI (Days 2, 5, 7, and 14), Among the Four Groups.

Group	Day 0		Day 2		Day 5		Day 7		Day 14	
	R	L	R	L	R	L	R	L	R	L
Group I	3.10 ± 0.32	3.10 ± 0.72	3.12 ± 0.29	4.89 ± 0.84	2.91 ± 0.46	4.10 ± 0.64	2.37 ± 0.34	4.78 ± 0.97	3.02 ± 0.63	4.36 ± 0.58
Group II	2.66 ± 0.35	2.89 ± 0.40	2.75 ± 0.48	5.35 ± 0.98	2.84 ± 0.28	4.95 ± 0.59	2.51 ± 0.51	6.40 ± 0.94	2.77 ± 0.41	4.98 ± 1.20
Group III	2.83 ± 0.45	2.81 ± 0.35	3.22 ± 0.50	3.23 ± 0.39	3.18 ± 0.41	3.23 ± 0.23	3.01 ± 0.53	3.08 ± 0.57	3.02 ± 0.43	3.05 ± 0.21
Group IV	3.00 ± 0.39	2.97 ± 0.33	2.99 ± 0.42	3.21 ± 0.40	3.18 ± 0.36	2.83 ± 0.29	2.92 ± 0.52	2.69 ± 0.42	3.02 ± 0.42	2.93 ± 0.54

R, OSOM of the right kidney; L, OSOM of the left kidney. Group I, IRI + MitoQ; Group II, IRI + saline; Group III, normal + MitoQ; Group IV, normal + saline.

**Figure 5:**
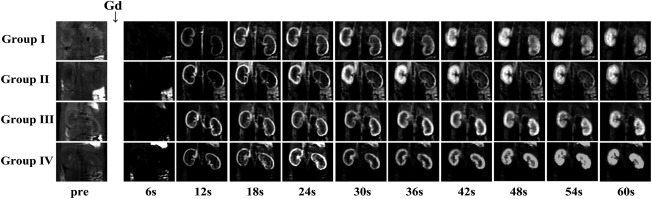
Examples of the dynamic contrast‐enhanced images of bilateral renal regions in the four groups on day 2 after IRI. On the precontrast images (pre), bilateral kidneys in four groups were seen with similar SI. After the administration of Gd‐DTPA (Gd ↓), the initial SI increase in the cortex was slower and lower in the left kidneys than in the right kidneys in Groups I and II, and the thicknesses of the cortex were thinner as well; however, the bilateral kidneys in Groups III and IV were enhanced to the same extent. The decrease of the left cortex enhancement was less obvious in Group I than in Group II. A clear corticomedullary boundary could be seen in all of the groups until 18 s. The renal medulla of the right kidneys in the four groups began to enhance at 24 s, after which the medulla achieved almost identical enchantment as the cortex between 48 and 60 s. However, there was a notable delay in the enhancement for the left kidneys in Groups I and II.

The DCE‐MRI showed that the initial SI increase in the cortex was slower and lower in the left kidneys than in the right kidneys in Groups I and II, and the thicknesses of the cortex were thinner as well; however, the bilateral kidneys in Groups III and IV were enhanced to the same extent. The decrease in the left cortex enhancement was less obvious in Group I than in Group II. A clear corticomedullary boundary could be seen in all the groups until 18 s. The renal medulla of the right kidneys in the four groups began to enhance at 24 s, and then the medulla achieved almost identical enhancement as the cortex between 48 and 60 s. However, there was a notable delay in the enhancement for the left kidneys in Groups I and II (Fig. 5).

**Figure 6:**
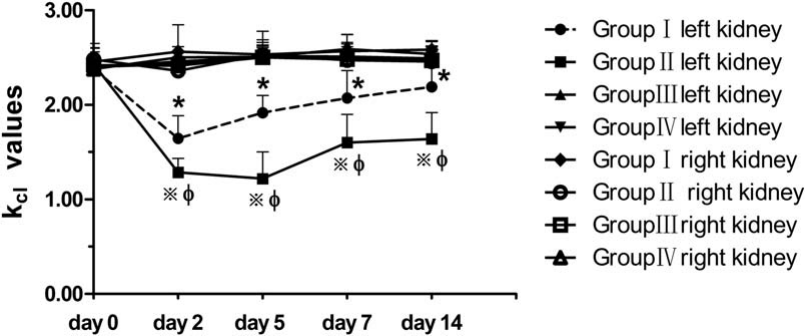
Graphs of the longitudinal changes of k_cl_ values on bilateral kidneys in the four groups before (day 0) and after IRI (days 2, 5, 7, and 14). K_cl_ values of the kidneys in all of the groups on day 0 were at approximately the same level. K_cl_ values of the left kidneys dropped dramatically on day 2 in Groups I and II, after which the values in Group I began to rise gradually but were still below the normal level; the values in Group II continued to drop until day 5, and they reached a much lower level than in Group I. K_cl_ values of the left kidneys in Groups I and II were lower than those of the other kidneys (the right kidneys in all of the groups and the left kidneys in Groups III and IV) after IRI. K_cl_ values of the left kidneys in Group I were higher than those in Group II at all time points after IRI (*P = * 0.002, *P < * 0.001, *P = * 0.001, *P < * 0.001). No significant differences in k_cl_ values existed between any two of the other kidneys. Significant differences of k_cl_ values between the left kidneys in Group I and the other kidneys are indicated as * (*P < * 0.05); those between the left kidneys in Group II and the other kidneys are indicated as ※ (*P < * 0.05); those between the left kidneys in Group I and the left kidneys in Group II are indicated as **φ** (*P < * 0.05).

The K_cl_ values for the four groups at different time points are listed in Table 2. K_cl_ values of the bilateral kidneys in the four groups on day 0 were not significantly different. K_cl_ values of the left kidneys dropped dramatically on day 2 in Groups I and II, after which the values in Group I began to rise gradually but were still below the normal level; meanwhile, the values in Group II continued to drop until day 5, and they reached a much lower level than in Group I. K_cl_ values of the left kidneys in Groups I and II were lower than those of the other kidneys (the right kidneys in all of the groups and the left kidneys in Groups III and IV) after IRI. K_cl_ values of the left kidneys in Group I were higher than those in Group II at all time points after IRI (*P* = 0.002, *P* < 0.001, *P* = 0.001, *P* < 0.001). No significant difference in k_cl_ values existed between any two of the other kidneys (Fig. 6).

**Table 2:** Average k_cl_ Values (Mean ± Standard Deviation) of the Bilateral Kidneys, Before (Day 0) and After IRI (Days 2, 5, 7, and 14), Among the Four Groups.

Group	Day 0		Day 2		Day 5		Day 7		Day 14	
	R	L	R	L	R	L	R	L	R	L
Group I	2.46 ± 0.10	2.38 ± 0.11	2.56 ± 0.28	1.64 ± 0.24	2.53 ± 0.25	1.92 ± 0.18	2.57 ± 0.18	2.07 ± 0.29	2.58 ± 0.09	2.19 ± 0.24
Group II	2.48 ± 0.17	2.43 ± 0.17	2.36 ± 0.12	1.29 ± 0.15	2.53 ± 0.08	1.22 ± 0.28	2.47 ± 0.08	1.60 ± 0.30	2.46 ± 0.10	1.64 ± 0.28
Group III	2.42 ± 0.18	2.40 ± 0.12	2.41 ± 0.09	2.43 ± 0.12	2.50 ± 0.13	2.52 ± 0.15	2.48 ± 0.13	2.51 ± 0.15	2.47 ± 0.11	2.49 ± 0.13
Group IV	2.38 ± 0.12	2.40 ± 0.07	2.50 ± 0.11	2.46 ± 0.09	2.51 ± 0.11	2.51 ± 0.18	2.59 ± 0.16	2.48 ± 0.16	2.54 ± 0.14	2.49 ± 0.12

R, right kidney; L, left kidney; Group I, IRI + MitoQ; Group II, IRI + saline; Group III, normal + MitoQ; Group IV, normal + saline.

**Figure 7:**
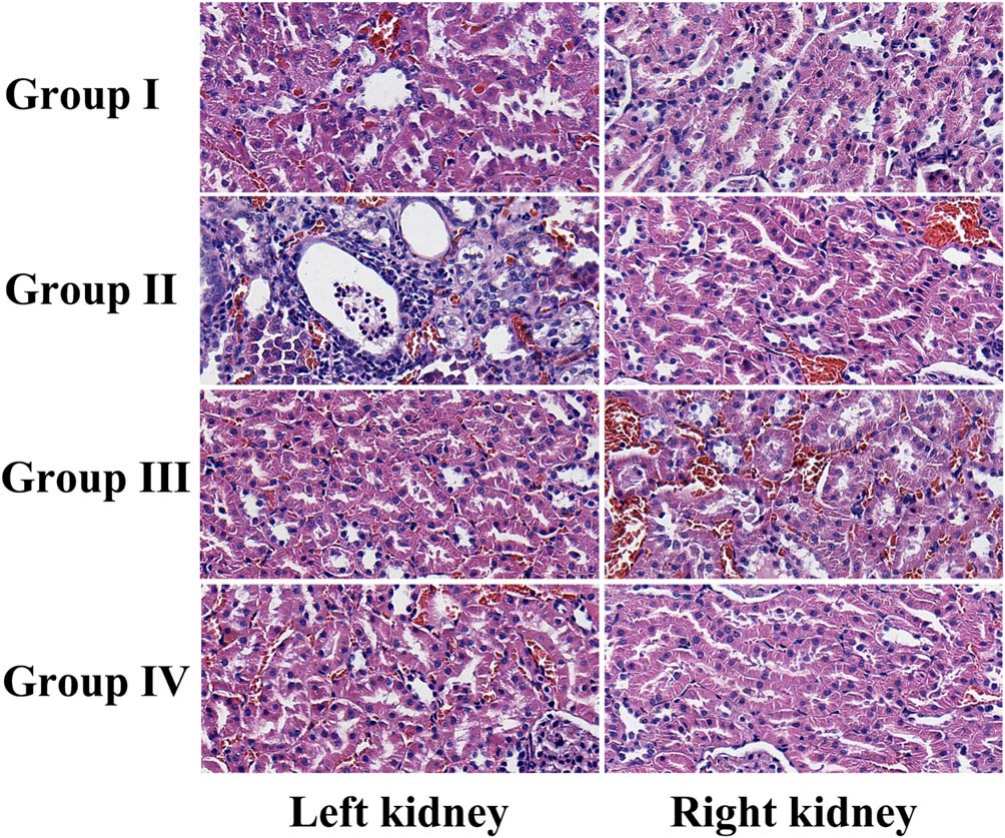
Microscopic specimens with hematoxylin and eosin staining ( × 400) of the bilateral kidneys on day 14 after IRI in the four groups. Tubular dilatation, tubular epithelial cell vacuolization, desquamation and cast formation, as well as interstitial tissue edema and inflammatory cell infiltration, were observed in the left kidneys in Group II, and they were less predominant in the left kidneys in Group I. The most severe lesions were observed in renal tubules and interstitial tissue located in the OSOM. Pathological changes in the other kidneys were minimal.

The renal tissue analysis involved alterations in renal tubules (tubular dilation, epithelial cell vacuolization, desquamation, and cast formation) and alterations in interstitial tissue (interstitial tissue edema and inflammatory cell infiltration), which were the most obvious in the OSOM of the left kidneys in Group II, and such ischemic tubulointerstitial abnormalities were less predominant in the left kidneys in Group I; the other kidneys exhibited minimal pathological changes (Fig. 7). Only the left kidneys in Group I and Group II demonstrated significantly increased renal damage scores compared with the other kidneys (*P* < 0.05 and *P* < 0.001, respectively), and the median renal damage score of the left kidneys in Group II was significantly higher than that in Group I (*P* < 0.001) (Table 3).

**Table 3:** Pathological Damage Scores (Median ± Q) of the Kidneys Among the Four Groups.

	Group I	Group II	Group III	Group IV
L	2.00 ± 0.25^a^, ^b^	4.00 ± 0.25^b^	1.00 ± 1.00	1.00 ± 1.00
R	1.00 ± 1.00	1.00 ± 1.00	1.00 ± 1.00	1.50 ± 1.00

L, left kidney; R, right kidney; Group I, IRI + MitoQ; Group II, IRI + saline; Group III, normal + MitoQ; Group IV, normal + saline.

a *P* < 0.05 compared with the left kidneys in Group II.

b *P* < 0.05 compared with the other kidneys.

## DISCUSSION

In this study, we showed that the mitochondria‐targeted antioxidant MitoQ could reduce the severity of renal damage caused by IRI using the longitudinal observation of T_2_‐weighted imaging and DCE‐MRI for the first time, which was confirmed by histopathological evaluation.

During renal IRI, the harmful hydrogen peroxide and ROS superoxide were critically increased from the mitochondria, causing damage to cellular lipids. Moreover, mitochondrial oxidative damage could destroy the ATP supply from mitochondria after reperfusion and lead to mitochondrial permeability transition pores and finally cell death 9. Growing evidence has shown the OSOM is sensitive to hypoperfusion 14, 22. The major damage occurs on proximal convoluted tubules, which are located on the OSOM: Ysebaert et al. reported that up to 80% of proximal tubular cells in the OSOM were extensively damaged within 12 h after clamping the renal artery for 60 min followed by reperfusion in a rat model 23. The increased SI of the OSOM on T_2_‐weighted imaging on IRI kidneys may be related to the increased tissue–water content caused by tissue edema and reperfusion. The renal histopathological findings in the present study also confirmed that the most severe lesions after IRI were observed in renal tubules and interstitial tissue located in the OSOM.

MitoQ reaches its maximum at 5 min in the kidney tissue after intravenous injection 24; thus, in this study, the injection via the tail vein 15 min before the beginning of ischemia could enable MitoQ to be taken up completely. Furthermore, the half‐life of MitoQ for the kidney is 4 h, and 80% of MitoQ could not be eliminated until 24 h after intravenous injection 24, which helps MitoQ work for several hours. The generation of lipid peroxyl radicals and enhanced ROS could be decreased within the mitochondria by MitoQ, preventing mitochondrial oxidative damage 11, which can explain the lower SSI and the higher k_cl_ values of IRI kidneys in the MitoQ pretreatment group than in the saline control group. However, MitoQ had little effect on healthy renal function (healthy kidneys with MitoQ pretreatment and saline control shared similar renal function parameters), which might be due to the normal balance between the cells' defense mechanism and the ROS production being maintained by the cellular redox homeostasis, whereas MitoQ worked only to lower the oxidative stress caused by the imbalance from the excessive production of ROS.

Noninvasive monitoring of the progression associated with renal IRI is of great importance 25. However, it is also a methodological challenge, because all the currently available techniques have limitations. Many researchers have monitored renal function with biochemical assays, such as serum creatinine levels, which could detect only the overall renal function of both kidneys 9, 26, 27. To recognize and monitor unilateral renal function, these studies had to use a rodent model subjected to contralateral nephrectomy 28, 29, 30, 31 or clamping of the bilateral renal arteries 32, 33, 34, 35, 36, which established a different pathological environment from that found in clinical practice. Moreover, repeatedly taking blood samples for longitudinal studies might interfere with the physiologic environment in a small animal model. Dynamic scintigraphic renography is ideal for measuring unilateral glomerular filtration rate 37; however, the method cannot be performed on rodent kidneys, as both the kidney and aorta are too small to be delineated.

Several mathematical models have been proposed to measure unilateral renal function from DCE‐MRI. Among of them, the Baumann‐Rudin model is unique because it does not depend on arterial input function, thus exempting the SI measurement error associated with motion and magnetic susceptible artifacts on the aorta or renal artery. This is a great advantage to be applied in rodent models, as their diameters are very small. The normal level of k_cl_ value (2.44 ± 0.14/min) in the present study differed from that of the literature published in the years 2000 and 2002, which ranged from 3.4 ± 0.5/min 16 to 1.53 ± 0.09/min 17. There may be several reasons for the discrepancy. First, the experiment in year 2002 was carried out on Wistar‐Kyoto rats. Second, the previous images were obtained with much longer acquisition times (over 10 s) and had poor spatial resolution.

There were several limitations in our study. First, the sample size was small; a larger sample study should be carried out for further investigation. In this way, dose‐dependent effects might be taken into consideration. Second, the renal systemic fibrosis induced by gadolinium was not taken into account. The current study aimed to evaluate MitoQ for its protective effect on renal function based on DCE‐MRI in the research field instead of necessarily suggesting that clinical patients undergo DCE‐MRI in practice. It would be interesting to determine whether noncontrast functional MRI could play a role in the evaluation of renal IRI, and this is an issue we would like to investigate in the future.

In conclusion, T_2_ signal intensities and k_cl_ values could demonstrate the presence of renal IRI, and MitoQ could reduce the severity of renal damage in rodent IRI models.
